# Risk and Protective Factors for Bullying at 11 Years of Age in a Spanish Birth Cohort Study

**DOI:** 10.3390/ijerph17124428

**Published:** 2020-06-19

**Authors:** Izaro Babarro, Ainara Andiarena, Eduardo Fano, Nerea Lertxundi, Martine Vrijheid, Jordi Julvez, Florencia B. Barreto, Serena Fossati, Jesus Ibarluzea

**Affiliations:** 1 Faculty of Psychology of the University of the Basque Country, 20018 Donostia/San Sebastian, Spain; ainara.andiarena@ehu.eus (A.A.); eduardo.fano@ehu.eus (E.F.); nerea.lertxundi@ehu.es (N.L.); flor.barreto@ehu.eus (F.B.B.); mambien3-san@euskadi.eus (J.I.); 2 Biodonostia Health Research Institute, Group of Environmental Epidemiology and Child Development, 20014 Donostia/San Sebastian, Spain; 3 Biomedical Research Centre Network for Epidemiology and Public Health (CIBERESP), 28029 Madrid, Spain; martine.vrijheid@isglobal.org (M.V.); jordi.julvez@isglobal.org (J.J.); 4 ISGlobal—Instituto de Salud Global de Barcelona, 08036 Barcelona, Spain; serena.fossati@isglobal.org; 5 Department of Experimental and Health Sciences, Universitat Pompeu Fabra, 08002 Barcelona, Spain; 6 Institut d’Investigació Sanitària Pere Virgili, Hospital Universitari Sant Joan de Reus, 43204 Reus, Spain; 7 Health Department of Basque Government, Sub-directorate of Public Health of Gipuzkoa, 20013 Donostia/San Sebastian, Spain

**Keywords:** bullying, children, prevalence, risk, individual, family, community and school factors

## Abstract

(1) Background: Bullying affects a large number of children worldwide. This study has two objectives, to provide data on the prevalence of bullying in Spain, and to identify risk and protective factors associated with bullying. (2) Methods: Participants were 858 eleven-year-old children. Bullying was assessed using a short version of the Olweus Bully Victim Questionnaire, and the following data were gathered to explore potential predictors: individual (inattention, behavior problems, attention deficit hyperactivity disorder symptomatology, traumatic life events), family-related (sociodemographic characteristics, family context, child-parent relations), school-related (school characteristics, peer and social support, school environment) and community-related data. (3) Results: 9.3% of the children were victims, 1.4% bullies and 1.6% bully-victims. Results showed that a higher level of attention deficit hyperactivity disorder symptomatology increased the risk of victimization, whereas having better relationships with parents and stronger social support were associated with a lower risk of victimization. Children having strong peer relationships and social support was also associated with less risk of perpetrating bullying. Finally, having behavior problems at 8 years of age was associated with being a bully-victim. (4) Conclusions: The findings emphasize the importance of studying all bullying predictors together, regarding three of the roles children may take in bullying situations.

## 1. Introduction

Bullying is defined as an aggressive behavior that happens in the school environment and is characterized by intentionality, repetitiveness and power imbalance between the bully and the victim [1]. It affects large numbers of children and adolescents worldwide, estimates indicating that between 8% [2] and 40% [3] of school students are involved in bullying. The variability in prevalence depends not only on the instrument used for evaluating the bullying, but also on the children’s sociocultural context. In a survey carried out between 2009 and 2010, assessing children in 38 European countries, the USA and Canada, the World Health Organization (WHO) found that the prevalence of bullying victimization ranged from 2% to 32%, and from 1% to 26% in the case of perpetration [4]. Large differences were also observed in the percentage of victimized children between European countries (Italy, England and Spain), in a cross-national European study by Ortega et al. (2012). Notably, their results showed that Spain has the lowest rates of victimization, particularly when talking about face-to-face bullying [5]. In Spain, García-García et al. (2017) found in their systematic review that the prevalence of bullying victimization was around 11.4% (range: 2.2%–29.01%) [6].

Due to the high prevalence and the impact it may have on people’s lives, it is important to study the protective and risk factors associated with bullying. Some systematic reviews and meta-analyses have concluded that certain individual, family, school and community factors are related to the involvement that a person may have in bullying situations [7,8,9,10,11,12].

Sex and age have been the most studied individual factors. Concerning sex, many researchers have observed a higher percentage of boys involved in bullying as a victim [13,14], as a bully [14,15,16,17] and as a bully-victim [17]. On the other hand, some studies have shown that being a girl increases the risk of experiencing psychological or general bullying victimization [3,18]. Regarding age, children are most likely to be bullied between 11–13 years [19], and from this age, the rates tend to decrease. Most studies have observed that younger age increases the risk of being involved in bullying [3,20,21,22], though there is evidence questioning this association [15].

Apart from sex and age, other individual factors have been studied in relation to bullying. For example, it has been seen that poor motor skills increase the risk of being a victim [17,23], while good ones decrease the risk of being involved as a bully [17]. Having a neurodevelopmental disorder has also been studied in relation to child involvement in bullying. Compared to children with typical development for their age, a higher percentage of children with intellectual disability or autism spectrum disorder are involved in bullying [24,25]. Moreover, children with poor executive function [26,27] or with emotional and behavior problems [3,21,28,29,30,31,32] have also been found to be more likely to be involved in bullying.

Furthermore, certain family characteristics have been classified as protective or risk factors for bullying. One of the most studied family factors in relation to bullying is family structure. Specifically, living with both parents has been identified as a protective factor for children’s involvement in bullying [14,30,33]. Parents’ socioeconomic level has also been investigated, it being found that low family income increases the risk of being involved in bullying [17,23,34,35,36,37]. Additionally, poor parental mental health has been linked to bullying involvement [30,38], as have traumatic or stressful life events in the family context, such as the death [16] or chronic illness of a family member [2], these increasing the risk of being involved as a bully or as a victim, respectively. Finally, a punitive parenting style [39] and family conflict [13,40] or violence [3,20,36,41,42] have also been related to increases in children´s bullying involvement.

Regarding school-related factors, having a good relationship with peers and teachers [14] reduces the risk of being involved in bullying. On the other hand, the perception of an inappropriate school climate [43], feeling a lack of safety at school [33,44], attending a public school [36,45] and large school size [21] increase the risk of being involved in bullying. Further, community factors, such as having problems with neighbors [28], concentrated poverty in the neighborhood and change of residence or residential instability [36] increase the risk of being involved in bullying, as a victim or a bully-victim.

Involvement in bullying situations affects children’s physical and psychological health. It has been found to be related to a wide range of problems, including poor mental health, substance abuse, somatic pain, being overweight or obese, poor academic achievement, loneliness [46], and even suicidal ideations [47]. This underlines the importance of identifying factors that increase the risk of being involved in bullying. To our knowledge, few empirical studies have analyzed the association individual, family, school and community predictors jointly have with bullying. And of these, only one focused on different roles that a child may have in bullying [13,33,36,48].

The present study has two objectives: first, to provide data on the prevalence of bullying in Spain based on the information provided by two cohorts of the INMA (INfancia y Medio Ambiente, from the Spanish for Children and the Environment, www.proyectoinma.org) project. Second, to identify individual, family, school and community related factors that may be associated with children´s involvement in bullying, considering three different roles: victim, bully and bully-victim.

## 2. Materials and Methods

### 2.1. Study Design and Participants

The study participants were children from the Gipuzkoa (Basque Country, Spain) and Sabadell (Catalonia, Spain) cohorts of the INMA project. This project collects data on children and their families in seven cohorts across Spain, and its main objective is to analyze the association between early exposure to environmental factors and children’s physical and neuropsychological development and health [49]. Participants´ mothers were informed about the INMA project and recruited in their first trimester of pregnancy in health centers or hospitals of the public health system. To be included, they were required to meet the following inclusion criteria: being older than 16 years old, having the intention of giving birth in their referral hospital, not having communication problems, having a single pregnancy and not having followed an assisted reproduction program. Since recruitment, data have been collected in several follow-up phases: in the first and third trimester of pregnancy, at birth, and when the child was 14 months, 26 months, 4 years, 8 years and 11 years of age. The ethical committees of the hospitals in the regions involved approved the project and informed consent has been obtained from all participants in each of the phases. In this study, we used data from the 8-years´ and 11-years´ follow-up phases. In the 11-years´ follow-up, 880 children and their families were visited and respondents were excluded from the present analysis if bullying items were missing (*n* = 22), yielding a final sample of 858.

### 2.2. Bullying

Bullying was assessed using a short version of the Olweus Bully Victim Questionnaire (OBVQ) [50] at the 11 years’ old follow-up, and children were asked to respond whilst thinking of the past 2 months. The OBVQ is a self-report instrument that has been widely used worldwide and which has shown satisfactory psychometric properties [51]. For the present study, we use a short version, which consists of a standardized definition of bullying and 16 questions. The first eight items refer to different victimization behaviors (physical, verbal, social, sexual and cyberbullying) and the second eight to physical, verbal, social, sexual or cyber harassment of another student. Items are rated on a 5-point Likert scale (0 “it hasn’t happened to me in the past couple of months”–4 “it happens several times a week”). The OBVQ showed adequate internal consistency in the present sample: α = 0.81 for the whole questionnaire, α = 0.82 for victim scale and α = 0.67 for bully scale. A dichotomized variable was created following the recommendations of Solberg and Olweus (2003). When participants answered “it happens 2 or 3 times a month” or more often to at least one of the items, they were categorized as victim, bully or bully-victim. 

### 2.3. Risk and Protective Factors at Different Follow up

#### 2.3.1. Eight-Year Follow-up

Strengths and Difficulties Questionnaire (SDQ) [52]: Parents were asked to complete the questionnaire to assess the general behavioral development of their children. The SDQ comprises 25 items in total, divided into 5 separate subscales: emotional symptoms, conduct problems, hyperactivity-inattention, peer relationship problems and prosocial behavior. The items are rated on a 3-point Likert scale (0 “not true”–2 “absolutely true”). In this study, the total difficulty score was used, which is generated by summing scores from all the scales except the prosocial scale, higher scores indicating more behavior problems. This questionnaire showed adequate psychometric properties in a Spanish sample [53] and the internal consistency for the SDQ was acceptable in the present sample: α = 0.78 for the total difficulty score used in the study.

Revised Conners’ Parent Rating Scale (CPRS-R)—Short form [54]: Parents completed the short form of the CPRS-R. This scale assesses problematic behavior in children and consists of 27 items rated on a 4-point Likert scale (0 “not true at all”–3 “very much true”) that yield scores for 3 subscales (Oppositional, Cognitive Problems/Inattention and Hyperactivity). For our study, we decided to use the ADHD index as a general measure of ADHD symptomatology. The Conners’ scales have been widely used and have shown adequate psychometric properties in a Spanish sample [55]. In the present sample, the CPRS-R showed adequate internal consistency: α = 0.92 for the ADHD index used in the study.

Haezi-Etxadi Family Assessment Scale 7-11 (HEFAS 7-11) [56]: Parents completed this instrument which assesses the quality of family context. It consists of 85 items divided into 5 subscales, namely: Promotion of cognitive and linguistic development, Promotion of socio-emotional development, Organization of the physical environment and social context, Parental stress and conflict, and Parental profile fostering child development. A higher score on the scale indicates a high quality of interactions in a family context. The psychometric properties of this scale are adequate [57] and in the INMA sample, the scale showed an acceptable internal consistency for each of the five subscales (α = 0.79, α = 0.83, α = 0.73, α = 0.75 and α = 0.80, respectively). This information was only collected in the Gipuzkoa cohort.

Attentional Network Task (ANT) [58,59]: This computerized task involves identifying the direction of the central arrow of a row of five arrows. Children are asked to press, as quickly as possible, the right or the left button, depending on the direction of the central arrow. The aim of this test is to assess the attention, alertness, orientation and conflict networks. It consists of 128 trials divided into 4 blocks. We used the hit reaction time standard error (HRT-SE), which is considered a measure of inattentiveness; a high HRT-SE indicates highly variable reactions.

Social cohesion and trust: Parents were asked to complete an ad-hoc questionnaire based on the Sampson et al. (1997) questionnaire [60]. It is composed of 4 questions rated on a 5-point Likert scale (0 “completely agree” to 4 “completely disagree”). A higher score on these questions indicate lower perceived social cohesion and trust in the neighborhood. In our sample, the questionnaire showed acceptable internal consistency: α = 0.79.

#### 2.3.2. Eleven-Year Follow-up

At the 11-year follow-up, in order to obtain repeated measures, we used some of the same questionnaires as in the earlier follow-up phase, namely, the SDQ, CPRS-R and ANT. We assessed the correlation between the repeated measures at 8 and 11 years of follow-up, obtaining moderate to high significant correlations for the repeated measures of the three variables: SDQ, CPRS-R and ANT. Hence, for these questionnaires, we decided to use the variable which required less transformation due to skewness. Specifically, data from the 8-year follow-up were used in the case of the SDQ, and from the 11-year follow-up in the case of CPRS-R and ANT.

In addition to these repeated measures, we used the following questionnaires:

Cups Task Roulette Version Test [61]: This is a computer task, consisting of 54 trials, that assesses decision making by observing the number of risky decisions a child makes. In this task, the participant is presented with two wheels divided into segments of equal size and each associated with an amount of money. On each trial, the participant is asked to choose which wheel to spin, in order to gain, or avoid losing, money. After the response, the wheel selected is spun for 2 s, and then ends on the amount of money to be won or lost. For this study, we took into account the total number of risky decisions each child made.

Questionnaire Kidscreen-27 [62]: This self-reported questionnaire consists of 27 items that are rated on a 5-point Likert scale (1 “not at all”–5 “very much”). The items are divided into five dimensions: Physical well-being, Psychological well-being, Peers and social support, Parents and autonomy, and School environment. In all cases, a higher score means a higher quality of the measured construct. The Spanish version of the Kidscreen-27 was validated, showing adequate psychometric properties [63,64]. The questionnaire showed acceptable internal consistency in the present sample for each of the subscales (α = 0.71 for physical wellbeing, α = 0.72 for psychological wellbeing, α = 0.73 for parents and autonomy, α = 0.73 for peer and social support and α = 0.70 for school environment).

Ad-hoc sociodemographic questionnaire: Parents were asked to complete a set of questions to gather data on family characteristics, including family structure (number of siblings, living with one or both parents), and parents´ age, educational level and social class. Parents were also asked about stressful family events since the birth of the child: change of residence, change of school, parental separation, death of a relative and hospitalization of a relative. On the other hand, school characteristics (type of school and number of students in the school) were obtained by asking the school principals.

Table 1 shows the main characteristics of the questionnaires and tests used in the present study.

### 2.4. Data Analysis

Statistical analyses were performed using SPSS v. 25. (IBM, Armonk, NY, USA). In the first step (exploratory data analysis), we studied the symmetry of each relevant variable, transforming data, when appropriate, using Tukey’s ladder of powers [65]. Further, we estimated the prevalence rates of bullying and measured the association between these and cohort and sex with Chi-square tests. In the second step, we applied logistic regression analysis [66], to build predictive models for the binary response variables: victim, bully, and bully-victim. In order to examine associations between the independent and dependent variables, bivariate analyses were performed using Chi-square test and independent t-tests. The models were constructed using potential predictors that were associated with the dependent variables at *p* < 0.10 in the bivariate analysis. After testing for marginal effects, in order to do a consistent selection of predictors variables, “forward selection” and “backward elimination” stepwise methods were used. The models were built using the selected variables and including cohort, sex and age, due to the study design and findings in the previous literature. Finally, sensitivity analyses were performed, because two predictive variables were only assessed in the Gipuzkoa sample.

## 3. Results

### 3.1. Sample Description

The study sample consisted of 858 children (51% girls and 49% boys) of 11 years (*M* = 10.94; *SD* = 0.49; *Min =* 9.54; *Max =* 12.86), from the INMA project cohorts of Gipuzkoa (*n*= 376) and Sabadell (*n* = 482) (Appendix A). The descriptive analysis showed no statistically significant differences between the cohorts by sex, but that the children from Sabadell were older, on average, than those from Gipuzkoa (*M* = 11.04; *SD* = 0.60; *M* = 10.83; *SD* = 0.24; *p* < 0.001).

### 3.2. Prevalence of Bullying

Results showed that 9.3% of the children (*n* = 80) were involved as a victim, 1.4% (*n* = 12) as a bully and 1.6% (*n* = 14) as a bully-victim. When exploring differences in prevalence between subgroups, the results showed no significant differences in prevalence by sex (Chi-square (3) = 5.31; *p* = 0.15) or cohort (Chi square (3) = 0.39; *p* = 0.94).

### 3.3. Bivariate Findings

The examination of bivariate relationships showed that the following potential predictors were associated at *p <* 0.10 with being involved as a victim (Appendix B): behavior problems at 8 years (measured with SDQ), ADHD symptomatology at 11 years (measured with the CPRS-R), inattention at 11 years (measured with the ANT), physical and psychological well-being, relationship with parents and autonomy, relationships with peers and social support at 11 years (all measured with Kidscreen-27), parental stress and conflict and parental profile fostering child development at 8 years (measured with HEFAS 7-11), parents’ social class, educational level, and availability of neighbors and trust in their neighborhood at 8 years.

As can be seen in Appendix C, only two variables were associated with being a bully (*p <* 0.10): peers and social support (measured with Kidscreen-27 at 11 years), and having had a family member hospitalized at any time in the child’s life.

The results of the bivariate analysis between the predictor variables and the involvement in bullying as a bully-victim (Appendix D) suggested that the variables associated (*p* < 0.10) with being a bully-victim were: children´s age and sex, behavior problems at 8-years’ follow-up (measured with the SDQ), ADHD symptomatology at 11-years´ follow-up (measured with the CPRS-R), inattention at 8 and 11-years’ follow-up (measured with the ANT), psychological well-being and school at 11-years’ follow-up (measured with Kidscreen-27), as well as the father´s social class, mother´s educational level and neighbors’ availability.

### 3.4. Logistic Regression Models

In this analysis, three models were built, one for each dependent variable (victim/not involved, bully/not involved, and bully-victim/not involved).

#### Victims: Predictor Variables

Binary logistic regression was carried out in order to explore the way in which individual-, family-, school- and community-related factors might predict the involvement of the children in bullying as a victim. The following variables were selected for inclusion in the model using forward and backward methods: ADHD symptomatology, parents and autonomy, and peers and social support. Finally, the model was adjusted for cohort sex and age (Table 2).

The model was statistically significant (*p* < 0.01) and explained 12.6% of the variance (R^2^ Nagelkerke = 0.126). Results showed that higher ADHD symptomatology increased the risk of being involved as a victim of bullying at 11 years (OR = 1.49; 95% CI = 1.22–1.82), while having greater autonomy and better relationships with parents (OR = 0.32; 95% CI = 0.16–0.66) and having stronger peer relationships and social support (OR = 0.99; 95% CI = 0.98–0.99) were related to a lower risk of being involved as a victim in bullying situations.

In addition, following the same method, a model was built for the Gipuzkoa sample separately, including the same variables and the score for family ecology at 8 years (Table 3).

This model was statistically significant (*p* < 0.01) and explained 11.3% of the variance (R^2^ Nagelkerke = 0.113). The results showed that having higher scores in family ecology, indicating lower levels of family stress and conflict, decreased the risk of being a victim of bullying (OR = 0.99, 95% CI = 0.99–0.99). Moreover, having a good relationship with parents (OR = 0.27; 95% CI = 0.09–0.80) was related to a lower risk of being a victim.

### 3.5. Bully: Predictor Variables

As can be seen in Appendix C, only two variables were associated with being a bully (*p <* 0.10): peers and social support and having had a family member hospitalized. As data on this latter variable were only collected for children in the Gipuzkoa cohort, we built one general model with the peers and social support variable, adjusted for cohort, sex and age (Table 4), and a different model for the Gipuzkoa sample (Table 5).

This model was not statistically significant (*p* = 0.07) and it explained 7.3% of the variance (R^2^ Nagelkerke = 0.073). The results suggested that having a good relationship with friends was associated with a lower risk of being a bully (OR = 0.97; 95% CI = 0.94–0.99).

In the case of the Gipuzkoa sample, the built model was statistically significant (*p =* 0.04) and it explained 17.4% of the variance (R^2^ Nagelkerke = 0.174). The results showed that having had a family member hospitalized increases children’s risk of being involved as a bully (OR = 7.32; 95% CI = 1.15–46.56).

### 3.6. Bully-Victim: Predictor Variables

As for the models of victims and bullies, variables were selected using forward and backward methods (inattention, behavior problems and school environment), and then the model was adjusted for cohort, sex and age (Table 6).

This model was statistically significant (*p <* 0.01) and it explained 20.6% of the variance (R^2^ Nagelkerke = 0.206). The results showed that the only variable significantly associated with being a bully-victim was having behavior problems at 8 years (OR = 2.58; 95% CI = 1.21–5.52), whereas having a good school environment was related to being involved in bullying as a bully-victim (OR = 0.68; 95% CI = 0.45–1.01).

## 4. Discussion

Concerning the prevalence of bullying, the overall rate of involvement in our study was of 12.3%. Breaking this rate down, 9.3% of the participants were victims, 1.4% bullies and 1.6% bully-victims. The prevalence of bullying varies depending on the sociocultural and socioeconomic context and the instruments used for the detection and evaluation of bullying. The WHO carried out a study between 2009 and 2010, evaluating the involvement in bullying (using an item based on the OBVQ) of 11- to 15-year-old children from 38 countries in Europe, the USA and Canada. Specifically, in the 11-year-olds, it was observed that on average 13% of the participants were victims of bullying, whereas the prevalence of bullies was 8% on average [4]. In the same study, data in Spanish children indicated that 4% of girls and 8% of boys were victims, while 3% of girls and 7% of boys were bullies [4]. Garcia-Garcia et al. (2017), in a systematic review, including 32 papers on Spanish samples, found that overall, on average, 11.4% (between 2% and 29.01% depending on the study) of students in Spain with a mean age of 14.60 (SD = 0.70) were involved in bullying situations [6]. Two papers on bullying in the same Spanish regions as our study reported similar prevalence data. Specifically, regarding the Basque country, it was found that 13.2% were victims, 1.6% bullies and 2% bully-victims [45], while in a study in Barcelona (i.e., the same province as our sample from Sabadell in Catalonia), it was found that 10.7% of children were involved in bullying [67]. Hence, our data are similar to the recent prevalence data for bullying in Spain. 

Although many previous studies have found that the involvement of preteens in bullying is sex dependent [14,15,18,22,34,36], in our study, we did not find a significant association between sex and the children’s involvement in bullying, although we did observe a slighter higher percentage of girls involved as a bully and higher percentage of boys involved as a bully-victim. Some other studies have also found no consistent association [2,68]. Another personal variable that has been widely studied in relation to bullying is age. Many researchers have shown a higher risk of being involved in bullying at younger ages [3,20,21,22,26]. Unlike several other studies, we did not find any significant associations between age and being involved as a victim, a bully or a bully-victim. This may be due to the design of our study, there being very small differences in age between the participants. 

Regarding the analysis of factors that could be associated with the involvement of boys and girls in bullying situations, in the case of the victims, we observed that more ADHD symptomatology as assessed with the CPRS-R at 11 years increases the risk of being a victim. Several studies have shown that children with behavior problems [69], such as externalizing problems [36], and more specifically, ADHD symptoms [70] or hyperactivity [29], have an increased risk of being involved in bullying situations as a victim. We also saw that a good relationship with friends and strong social support, as assessed with Kidscreen-27, decrease the risk of being involved in bullying situations as a victim at 11 years. Other researchers have found that a child having trust in school [71], a good relationship with classmates [14] and stronger peer and social support [72] decreased the risk of being involved in bullying situations. In the case of participants from Gipuzkoa, the sensitivity analysis showed that including family ecology (as assessed with HEFAS 7-11) at 8 years, the associations of bullying victimization with ADHD symptoms and with peer and social support found previously became non-significant. In addition, by including these factors, the variance explained by the model changed from 12.6% to 11.3%. Specifically, having greater stress and family conflict at 8 years increased the risk of being involved in bullying situations as a victim; on the other hand, a better perceived relationship with parents decreases the risk of being involved in bullying situations. Some studies have shown that good connectivity, understanding on the part of parents and good communication between parents and children are associated with decreases in the risk of being involved in situations of bullying [30,72], while family conflict increases the risk [13,40]. 

In the case of predictors of being a bully, we calculated two models, because the data about whether someone in the family had been hospitalized were only collected for participants from the Gipuzkoa cohort. As for being a victim, the relationship with peers and social support received from them, as assessed with Kidscreen-27, reduced the risk of being involved in bullying situations as a bully. On the other hand, having experienced the hospitalization of a relative increases the risk of being a bully. In line with this, a previous study found that having a family member with a chronic disease increased children’s risk of being a bully [2]. However, having a family member hospitalized was the unique of the studied stressful life events which showed an association with the implication children have in bullying. This could be due to the fact that our questions about stressful life events referred to the complete life of children rather than to a close period of their involvement in bullying.

Finally, the model for being a bully-victim showed that having behavior problems at 8 years (as assessed with SDQ) increased the risk of being involved in bullying. In line with this, symptoms of externalizing problems [28] in general, and of ADHD [32] in particular, have been associated with an increased risk of being involved in situations of bullying as a bully-victim.

### Study Limitations and Strengths

This study is not without limitations. First, data on bullying were collected using a self-report and non-validated questionnaire; and second, compared to individual and family-related factors, relatively few school- and community-related factors have been considered. Third, despite the prevalence we observed being highly consistent with data from previous studies, our sample is relatively small for estimating the prevalence in Spain and it may not be representative, in that it only takes into account data from participants located in two geographical areas: Gipuzkoa and Catalonia. Moreover, it should be pointed out that few children were identified to be involved in bullying as a bully or a bully-victim, thus, the results obtained should be treated with caution. Finally, although we analyzed the impact behavior problems in general, and that ADHD, in particular, could have in bullying, we did not study other psychopathological dimensions, such as autism, which has been related to bullying in previous literature. Finally, the model for being a bully-victim showed that having behavior problems at 8 years (as assessed with SDQ) increased the risk of being involved in bullying. In line with this, symptoms of externalizing problems [28] in general, and of ADHD [32] in particular, have been associated with an increased risk of being involved in situations of bullying as a bully-victim. 

Nevertheless, it should be highlighted that, to our knowledge, this is the first study in Spain that analyzes the association that individual, family, school and community predictors have with bullying, taking into account three of the roles children could take in bullying (victims, bullies and bully-victims). This is interesting, as it makes it possible to explore the way in which different factors affecting an individual may have an impact on the occurrence of particular events or development of a behavior like bullying. In addition, taking into account data from two follow-ups may provide clues as to who may be most at risk of being involved in this kind of situation, in relation to an individual´s family, social and school environment at an early age. Such information could help guide prevention programs, by identifying at-risk individuals. For future research, it would be desirable to continue studying the predictive factors together, analyzing the mediation and moderation effects of the different factors on participants who take different roles in bullying. 

## 5. Conclusions

In our study, carried out with two cohorts of Spanish children and their families, the rate of bullying victimization was 9.3%, while 1.4% of the children were bullies and 1.6% bully-victims. In general, results indicate a considerable role of a child´s social skills, behavioral patterns, peer and family relationships in bullying situations. Our findings underline the importance of studying all influences on bullying together, and that identifying the factors associated with bullying might facilitate the prevention of bullying in at-risk children.

## Figures and Tables

**Table 1:** Summary of instruments used.

Instrument	Type of Instrument	What is Assessed?	Reported by	Follow-up
**Individual Predictors**				
Strengths and Difficulties Questionnaire	Questionnaire	Behavior problems	Parents	8 years
Conners’ Parent Rating Scale	Questionnaire	ADHD symptomatology	Parents	11 years
Attentional Network Task	Neuropsychological test	Attention	Children	11 years
Cups Task, Roulette Version	Neuropsychological test	Executive function	Children	11 years
Kidscreen-27: Physical and psychological well-being	Questionnaire	Level of physical activity, energy and fitness, and positive emotions and satisfaction with life	Children	11 years
Life stressful events	Questionnaire	Stressful events in the course of the child’s life	Parents	11 years
**Family predictors**				
Haezi-Etxai Family Assessment Scale 7-11	Questionnaire	Family context	Parents	8 years
Kidscreen-27: parents and autonomy.	Questionnaire	Child’s interaction with parents, autonomy, financial resources	Children	11 years
**School and community predictors**				
Kidscreen-27: peers and social support	Questionnaire	Child’s relationship with friends and support received from them	Children	11 years
Kidscreen-27: school environment	Questionnaire	Child’s relationship teachers and feelings about school	Children	11 years
Social cohesion and trust	Questionnaire	Social cohesion and trust	Parents	11 years

**Table 2:** Predictors of being a victim for the whole sample.

Variable	B	SE	*p*	OR	CI 95%	
Constant	−0.07	3.01	0.98	0.93		
Cohort: Gipuzkoa	0.21	0.26	0.42	1.24	0.74	2.07
Age	−0.17	0.27	0.54	0.85	0.50	1.44
Sex: Girl	0.06	0.26	0.82	1.06	0.63	1.79
ADHD symptomatology (Revised Conners’ Parent Rating Scale) at 11-years’ follow-up	0.40	0.10	0.00	1.49	1.22	1.82
Parents and autonomy (Kidscreen-27) at 11-years’ follow-up	−1.14	0.37	0.00	0.32	0.16	0.66
Peers and social support (Kidscreen-27) at 11-years’ follow-up	−0.01	0.01	0.00	0.99	0.98	0.99

Notes: B = beta; SE = standard error; OR = odds ratio; CI = confidence interval.

**Table 3:** Predictors of being a victim for the Gipuzkoa cohort.

Variable	B	SE	*p*	OR	CI 95%	
Constant	−7.23	9.01	0.42	0.00		
Age	0.69	0.82	0.40	1.99	0.40	9.97
Sex: Girl	0.04	0.40	0.93	1.04	0.47	2.29
ADHD symptomatology (Revised Conners’ Parent Rating Scale) at 11-years’ follow-up	0.28	0.15	0.07	1.32	0.98	1.78
Parents and autonomy (Kidscreen-27) at 11-years’ follow-up	−1.31	0.56	0.02	0.27	0.09	0.80
Peers and social support (Kidscreen-27) at 11-years’ follow-up	0.00	0.01	0.96	1.00	0.99	1,02
Parental stress and conflict (Haezi Etxadi Family Assesment Scale 7-11) at 8-years’ follow up	0.00	0.00	0.05	0.99	0.99	0.99

Notes: B = beta; SE = standard error; OR = odds ratio; CI = confidence interval.

**Table 4:** Predictors of being a bully for the whole sample.

Variable	B	SE	*p*	OR	CI 95%	
Constant	−6.57	7.13	0.36	0.00		
Cohort: Gipuzkoa	0.21	0.62	0.73	1.24	0.37	4.15
Age	0.22	0.64	0.73	1.24	0.36	4.37
Sex: Girl	0.80	0.63	0.20	2.23	0.65	7.72
Peers and social support (Kidscreen-27) at 11-years’ follow-up	−0.03	0.01	0.02	0.97	0.94	0.99

Notes: B = beta; SE = standard error; OR = odds ratio; CI = confidence interval.

**Table 5:** Predictors of being a bully for the Gipuzkoa sample.

Variable	B	SE	*p*	OR	CI 95%	
Constant	−18.85	17.68	0.29	0.00		
Age	1.28	1.61	0.43	3.58	0.15	83.35
Sex: Girl	1.93	1.16	0.09	6.92	0.71	67.37
Peers and social support (Kidscreen-27) at 11-years’ follow-up	−0.05	0.04	0.16	0.95	0.88	1.02
Hospitalization of a family member	1.99	0.94	0.04	7.32	1.15	46.56

Notes: *B =* beta; *SE* = standard error; *OR =* odds ratio; *CI =* confidence interval.

**Table 6:** Predictors of being a bully-victim for the whole sample.

Variable	B	SE	*p*	OR	CI 95%	
Constant	4.00	8.15	0.62	54.58		
Cohort: Gipuzkoa	0.55	0.62	0.37	1.74	0.52	5.80
Age	−1.23	0.73	0.09	0.29	0.07	1.22
Sex: Girl	−0.31	0.65	0.63	0.73	0.21	2.60
Inattention (Attentional Network Task) at 11 years’ follow up	0.19	0.11	0.08	1.21	0.98	1.50
Behavior problems (Strengths and Difficulties Questionnaire) at 8 years’ follow up	0.95	0.39	0.02	2.58	1.21	5.52
School environment (Kidscreen-27) at 11 years’ follow-up	−0.39	0.20	0.05	0.68	0.45	1.01

Notes: B = beta; SE = standard error; OR = odds ratio; CI = confidence interval.

## Appendix A

**Table A1:** Description of the Sample.

Variable		%	Mean (SD)
**Individual variables**			
Sex	Female	51%	
	Male	49%	
Age			10.94 (0.49)
Behavior problems at 8 y (SDQ)			2.79 (0.91)
Inattention at 11 y (ANT)			13.70 (2.74)
ADHD symptomatology at 11y (CPRS-R)			2.38 (1.32)
Risky decisions at 11 y (Cups Task)			30.39 (9.28)
Phisical Wellbeing at 11 y (Kidscreen-27)			1.21 (0.44)
Psychologycal Wellbeing at 11 y (Kidscreen-27)			1.57 (0.39)
**Family variables**			
Mother age			43.19 (3.80)
Father age			45.29 (4.60)
Mother´s social class	Manual	35.6%	
	No manual	55.2%	
Father´s social class	Manual	57.4%	
	No manual	37.4%	
Mother´s study level	Primary	18.8%	
	Scondary	39.2%	
	Universitary	42%	
Father´s study level	Primary	29.9%	
	Scondary	42.9%	
	Universitary	27.2%	
Number of siblings			1.09 (0.51)
To be de oldest sibling	No	41.2%	
	Yes	58.8%	
Live with both parents	No	17.4%	
	Yes	82.6%	
Promotion of cognitive and linguistic development (HEFAS 7-11) at 8 y			67.02 (11.38)
Promotion of socio-emotional development (HEFAS 7-11) at 8 y			79.46 (7.84)
Organization of the physical environment and social context (HEFAS 7-11) at 8 y			86.74 (6.79)
Parental stress and conflict (HEFAS 7-11) at 8 y			77.26 (9.41)
Parental profile fostering child development (HEFAS 7-11) at 8 y			79.36 (8.79)
Change of residence	No	88.7%	
	Yes	11.3%	
Chage of school	No	96.5%	
	Yes	3.5%	
Parental separation	No	92.2%	
	Yes	7.8%	
Death of a relative	No	64.1%	
	Yes	35.9%	
Hospitalization of a relative	No	90.6%	
	Yes	9.4%	
Autonomy and parents at 11y (Kidscreen-27)			1.20 (0.37)
**School and community variables**			
Peers and social support at 11y (Kidscreen-27)			30.49 (30.23)
School environment at 11y (Kidscreen-27)			2.70 (1.37)
Type of school	Private	39.5%	
	Public	60.5%	
Stimate number of students in the school			586.69 (278.29)
A place the parents enjoy living in			1.77 (0.95)
It is easy to get practical help from neighboors			2.10 (0.91)
Most people can be trusted in the neighborhood			2.22 (0.97)
There are people I can turn to for advise			2.05 (0.94)

## Appendix B

**Table A2:** Bivariate Analysis for Being a Victim.

Variable		Víctim	Not Involved	Víctim	Not Involved	*p Value*
		N (%)		Mean (SD)		
**Individual variables**						
Sex	Female	36 (45%)	402 (51.7%)			*p* = 0.26
	Male	44 (55%)	376 (48.3%)			
Age				10.90 (0.48)	10.95 (0.49)	*p* = 0.41
Behavior problems at 8 y (SDQ)				3.01 (0.85)	2.76 (0.91)	*p* = 0.02
Inattention at 11 y (ANT)				14.28 (2.78)	13.64 (2.73)	*p =* 0.05
ADHD symptomatology at 11y (CPRS-R)				3.03 (1.45)	2.32 (1.29)	*p =* 0.00
Risky decisions at 11 y (Cups Task)				31.58 (7.58)	30.27 (9.43)	*p =* 0.24
Phisical Wellbeing at 11 y (Kidscreen-27)				1.066 (0.43)	1.23 (0.44)	*p =* 0.00
Psychologycal Wellbeing at 11 y (Kidscreen-27)				1.38 (0.43)	1.59 (0.38)	*p =* 0.00
**Family variables**						
Mother age				42.61 (4.23)	43.25 (3.75)	*p =* 0.15
Father age				45.64 (5.27)	45.26 (4.53)	*p =* 0.49
Mother´s social class	Manual	38 (51.4%)	275 (37.9%)			*p =* 0.02
	No manual	36 (48.6%)	450 (62.1%)			
Father´s social class	Manual	48 (66.7%)	396 (56.5%)			*p =* 0.09
	No manual	24 (33.3%)	305 (43.5%)			
Mother´s study level	Primary	24 (30.4%)	135 (17.6%)			*p =* 0.02
	Scondary	27 (34.2%)	304 (39.7%)			
	Universitary	28 (35.4%)	327 (42.7%)			
Father´s study level	Primary	24 (34.8%)	209 (29.4%)			*p =* 0.49
	Scondary	30 (43.5%)	304 (42.8%)			
	Universitary	15 (21.7%)	197 (27.7%)			
Number of siblings				1.17 (0.51)	1.08 (0.51)	*p =* 0.30
To be de oldest sibling	No	11 (31.4%)	132 (42.3%)			*p =* 0.22
	Yes	24 (68.6%)	180 (57.7%)			
Live with both parents	No	16 (20.3%)	131 (17.1%)			*p =* 0.48
	Yes	63 (79.7%)	635 (82.9%)			
Promotion of cognitive and linguistic development (HEFAS 7-11) at 8 y				66.71 (9.32)	67.05 (11.60)	*p =* 0.87
Promotion of socio-emotional development (HEFAS 7-11) at 8y				78.17 (8.74)	79.60 (7.74)	*p =* 0.31
Organization of the physical environment and social context (HEFAS 7-11) at 8y				85.07 (7.92)	86.93 (6.63)	*p =* 0.13
Parental stress and conflict (HEFAS 7-11) at 8y				72.94 (8.14)	77.74 (9.43)	*p =* 0.00
Parental profile fostering child development (HEFAS 7-11) at 8y				74.99 (12.08)	79.85 (8.22)	*p =* 0.002
Change of residence	No	34 (97.1%)	297 (87.9%)			*p =* 0.99
	Yes	1 (2.9%)	41 (12.1%)			
Chage of school	No	33 (94.3%)	327 (96.7%)			*p =* 0.45
	Yes	2 (5.7%)	11 (3.3%)			
Parental separation	No	30 (85.7%)	314 (92.9%)			*p =* 0.13
	Yes	5 (14.3%)	24 (7.1%)			
Death of a relative	No	23 (65.7%)	216 (63.9%)			*p =* 0.83
	Yes	12 (34.3%)	122 (36.1%)			
Hospitalization of a relative	No	31 (88.6%)	307 (90.8%)			*p =* 0.66
	Yes	4 (11.4%)	31 (9.2%)			
Autonomy and parents at 11y (Kidscreen-27)				0.99 (0.39)	1.22 (0.36)	*p =* 0.00
**School and community variables**						
Peers and social support at 11y (Kidscreen-27)				17.10 (25.15)	31.86 (30.86)	*p =* 0.00
School environment at 11y (Kidscreen-27)				1.92 (1.42)	2.78 (1.34)	*p =* 0.00
Type of school	Private	24 (36.9%)	274 (39.8%)			*p =* 0.65
	Public	41 (63.1%)	415 (60.2%)			
Stimate number of students in the school				537.27 (228.207)	591.33 (282.23)	*p =* 0.14
A place the parents enjoy living in				1.84 (1.11)	1.80 (0.94)	*p =* 0.74
It is easy to get practical help from neighboors				2.15 (1.09)	2.11 (0.92)	*p =* 0.69
Most people can be trusted in the neighborhood				2.48 (1.22)	2.21 (0.98)	*p =* 0.02
There are people I can turn to for advise				2.27 (1.19)	2.04 (0.94)	*p =* 0.05

## Appendix C

**Table A3:** Bivariate Analysis for Being a Bully.

Variable		Bully	Not Involved	Bully	Not Involved	*p Value*
		N (%)		Mean (SD)		
**Individual variables**						
Sex	Female	8 (66.7%)	426 (50.7%)			*p =* 0.27
	Male	4 (33.3%)	415 (49.3%)			
Age				10.97 (0.30)	10.94 (0.49)	*p =* 0.17
Behavior problems at 8 y (SDQ)				2.80 (1.14)	2.79 (0.90)	*p =* 0.98
					
Inattention at 11 y (ANT)				13.92 (2.87)	13.69 (2.75)	*p =* 0.78
ADHD symptomatology at 11y (CPRS-R)				2.17 (1.25)	2..38 (1.32)	*p =* 0.59
Risky decisions at 11 y (Cups Task)				32.36 (8.27)	30.35 (9.29)	*p =* 0.48
Phisical Wellbeing at 11 y (Kidscreen-27)				1.14 (0.56)	1.21 (0.44)	*p =* 0.60
Psychologycal Wellbeing at 11 y (Kidscreen-27)				1.44 (0.47)	1.58 (0.39)	*p =* 0.38
**Family variables**						
Mother age				43.64 (3.59)	43.18 (3.80)	*p =* 0.68
Father age				44.54 (4.52)	45.31 (4.58)	*p =* 0.56
Mother´s social class	Manual	3 (27.3%)	310 (39.4%)			*p =* 0.41
	No manual	8 (72.7%)	476 (60.6%)			
Father´s social class	Manual	7 (63.6%)	436 (57.4%)			*p =* 0.68
	No manual	4 (36.4%)	324 (42.6%)			
Mother´s study level	Primary	1 (8.3%)	158 (19.1%)			*p =* 0.63
	Scondary	5 (41.7%)	323 (39%)			
	Universitary	6 (50%)	347 (41.9%)			
Father´s study level	Primary	3 (27.3%)	228 (29.8%)			*p =* 0.79
	Scondary	4 (36.4%)	330 (43.1%)			
	Universitary	4 (36.4%)	207 (27.1%)			
Number of siblings				1.00 (0.71)	1.09 (0.51)	*p =* 0.71
To be de oldest sibling	No	3 (60%)	140 (40.9%)			*p =* 0.39
	Yes	2 (40%)	202 (59.1%)			
Live with both parents	No	2 (16.7%)	142 (17.1%)			*p =* 0.97
	Yes	10 (83.3%)	686 (82.9%)			
Promotion of cognitive and linguistic development (HEFAS 7-11) at 8 y				71.21 (7.42)	66.96 (11.42)	*p =* 0.41
Promotion of socio-emotional development (HEFAS 7-11) at 8y				77.82 (7.47)	79.48 (7.86)	*p =* 0.64
Organization of the physical environment and social context (HEFAS 7-11) at 8y				84.12 (8.67)	86.78 (6.76)	*p =* 0.39
Parental stress and conflict (HEFAS 7-11) at 8y				73.33 (9.69)	77.31 (9.41)	*p =* 0.35
Parental profile fostering child development (HEFAS 7-11) at 8y				74.29 (5.46)	79.43 (8.81)	*p =* 0.19
Change of residence	No	6 (100%)	325 (88.6%)			*p =* 0.38
	Yes	0 (0%)	42 (11.4%)			
Chage of school	No	6 (100%)	354 (96.5%)			*p =* 0.64
	Yes	0 (0%)	13 (3.5%)			
Parental separation	No	6 (100%)	338 (92.1%)			*p =* 0.47
	Yes	0 (0%)	29 (7.9%)			
Death of a relative	No	3 (50%)	236 (64.3%)			*p =* 0.47
	Yes	3 (50%)	131 (35.7%)			
**Hospitalization of a relative**	No	4 (66.7%)	334 (91%)			*p =* 0.04
	Yes	2 (33.3%)	33 (9%)			
Autonomy and parents at 11y (Kidscreen-27)				1.02 (0.37)	1.20 (0.37)	*p =* 0.12
**School and community variables**						
Peers and social support at 11y (Kidscreen-27)				10.25 (8.51)	30.89 (30.37)	*p =* 0.02
School environment at 11y (Kidscreen-27)				2.49 (1.41)	2.71 (1.37)	*p =* 0.58
Type of school	Private	4 (36.4%)	293 (39.6%)			*p =* 0.83
	Public	7 (63.6%)	447 (60.4%)			
Stimate number of students in the school				579.73 (220.35)	587.43 (279.55)	*p =* 0.93
A place the parents enjoy living in				1.44 (0.53)	1.81 (0.96)	*p =* 0.26
It is easy to get practical help from neighboors				2.33 (1.12)	2.11 (0.94)	*p =* 0.47
Most people can be trusted in the neighborhood				2.56 (1.014)	2.24 (1.01)	*p =* 0.34
There are people I can turn to for advise				2.33 (1.00)	2.06 (0.97)	*p =* 0.39

## Appendix D

**Table A4:** Bivariate Analysis for Being a Bully-Victim.

Variable		Bully-Victim	Not Involved	Bully-Víctim	Not Involved	*p Value*
		N (%)		Mean (SD)		
**Individual variables**						
Sex	Female	4 (28.6%)	430 (51.3%)			*p =* 0.09
	Male	10 (71.4%)	409 (28.7%)			
Age				10.69 (0.43)	10.95 (0.49)	*p =* 0.05
Behavior problems at 8 y (SDQ)				3.65 (0.51)	2.78 (0.90)	*p =* 0.00
Inattention at 11 y (ANT)				15.56 (3.29)	13.66 (2.73)	*p =* 0.01
ADHD symptomatology at 11y (CPRS-R)				3.04 (1.08)	2.37 (1.32)	*p =* 0.05
Risky decisions at 11 y (Cups Task)				31.21 (5.21)	30.36 (9.33)	*p =* 0.73
Phisical Wellbeing at 11 y (Kidscreen-27)				1.15 (0.34)	1.21 (0.44)	*p =* 0.62
Psychologycal Wellbeing at 11 y (Kidscreen-27)				1.26 (0.33)	1.58 (0.39)	*p =* 0.00
**Family variables**						
Mother age				42.41 (4.16)	43.20 (3.79)	*p =* 0.44
Father age				44.42 (3.10)	45.31 (4.60)	*p =* 0.47
Mother´s social class	Manual	6 (54.5%)	307 (39.1%)			*p =* 0.30
	No manual	5 (45.5%)	479 (60.9%)			
Father´s social class	Manual	10 (90.9%)	433 (57%)			*p =* 0.02
	No manual	1 (9.1%)	327 (43%)			
Mother´s study level	Primary	6 (42.9%)	153 (18.5%)			*p =* 0.06
	Scondary	3 (21.4%)	325 (39.3%)			
	Universitary	5 (35.7%)	348 (42.1%)			
Father´s study level	Primary	8 (61.5%)	223 (29.2%)			*p =* 0.33
	Scondary	4 (30.8%)	330 (43.3%)			
	Universitary	1 (7.7%)	210 (27.5%)			
Number of siblings				1.00 (0.63)	1.09 (0.51)	*p =* 0.68
To be de oldest sibling	No	3 (50%)	140 (41.1%)			*p =* 0.66
	Yes	3 (50%)	201 (58.9%)			
Live with both parents	No	1 (7.1%)	143 (17.3%)			*p =* 0.32
	Yes	13 (92.9%)	683 (82.7%)			
Promotion of cognitive and linguistic development (HEFAS 7-11) at 8 y				66.41 (9.21)	67.03 (11.42)	*p =* 0.90
Promotion of socio-emotional development (HEFAS 7-11) at 8y				79.10 (10.20)	79.46 (7.82)	*p =* 0.91
Organization of the physical environment and social context (HEFAS 7-11) at 8y				86.60 (7.74)	86.74 (6.78)	*p =* 0.96
Parental stress and conflict (HEFAS 7-11) at 8y				74.77 (10.37)	77.30 (9.40)	*p =* 0.51
Parental profile fostering child development (HEFAS 7-11) at 8y				77.38 (8.38)	79.39 (8.80)	*p =* 0.58
Change of residence	No	7 (100%)	324 (88.5%)			*p =* 0.34
	Yes	0 (100%)	42 (11.5%)			
Chage of school	No	7 (100%)	353 (96.4%)			*p =* 0.61
	Yes	0 (0%)	13 (3.6%)			
Parental separation	No	7 (100%)	337 (92.1%)			*p =* 0.44
	Yes	0 (0%)	29 (7.9%)			
Death of a relative	No	5 (71.4%)	234 (63.9%)			*p =* 0.68
	Yes	2 (28.6%)	132 (36.1%)			
Hospitalization of a relative	No	6 (85.7%)	332 (90.7%)			*p =* 0.65
	Yes	1 (14.3%)	34 (9.3%)			
Autonomy and parents at 11y (Kidscreen-27)				1.08 (0.31)	1.20 (0.37)	*p =* 0.22
**School and community variables**						
Peers and social support at 11y (Kidscreen-27)				27.37 (32.95)	30.65 (30.24)	*p =* 0.69
School environment at 11y (Kidscreen-27)				1.78 (1.42)	2.72 (1.36)	*p =* 0.01
Type of school	Private	6 (60%)	291 (39.3%)			*p =* 0.18
	Public	4 (40%)	450 (60.7%)			
Stimate number of students in the school				575.5 (115.42)	587.48 (280.25)	p = 0.89
A place the parents enjoy living in				2.17 (1.12)	1.80 (0.96)	*p =* 0.19
It is easy to get practical help from neighboors				2.58 (1.24)	2.10 (0.93)	*p =* 0.08
Most people can be trusted in the neighborhood				2.00 (0.60)	2.24 (1.01)	*p =* 0.41
There are people I can turn to for advise				2.33 (1.16)	2.06 (0.96)	*p =* 0.33
